# *N*-[4-Acetyl-5-(3-methoxy­phen­yl)-4,5-dihydro-1,3,4-thia­diazol-2-yl]acetamide

**DOI:** 10.1107/S1600536808032108

**Published:** 2008-10-11

**Authors:** G. Aridoss, S. Amirthaganesan, D. Velmurugan, S. H. Kim, Y. T. Jeong

**Affiliations:** a Division of Image and Information Engineering, Pukyong National University, Busan 608-739, Republic of Korea; b Centre of Advanced Study in Crystallography and Biophysics, University of Madras, Guindy Campus, Chennai 600 025, India

## Abstract

The title compound, C_13_H_15_N_3_O_3_S, crystallizes with two mol­ecules in the asymmetric unit. The thia­diazole rings in both the mol­ecules adopt an envelope conformation. The crystal packing is stabilized by inter­molecular N—H⋯O and C—H⋯O inter­actions.

## Related literature

For biological activities of thia­diazole derivatives, see: Balasubramanian *et al.* (2004 ▶); Li *et al.* (2001 ▶); Radwan *et al.* (2007 ▶); Supuran *et al.* (2001 ▶). For ring conformational analysis, see: Cremer & Pople (1975 ▶); Nardelli (1983 ▶).
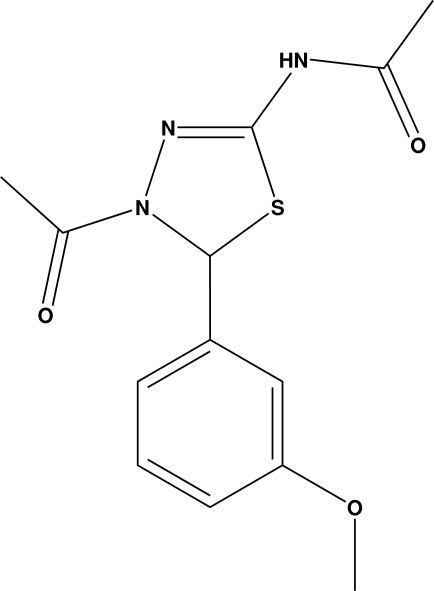


## Experimental

### 

#### Crystal data

C_13_H_15_N_3_O_3_S
*M* *_r_* = 293.34
Monoclinic, 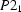

*a* = 11.3790 (4) Å
*b* = 10.5993 (3) Å
*c* = 11.9596 (2) Å
β = 108.225 (2)°
*V* = 1370.08 (7) Å^3^
*Z* = 4
Mo *K*α radiation
μ = 0.25 mm^−1^
*T* = 293 (2) K
0.30 × 0.20 × 0.16 mm

#### Data collection

Bruker Kappa APEXII CCD diffractometer
Absorption correction: multi-scan (*SADABS*; Bruker, 1999 ▶) *T*_min_ = 0.930, *T*_max_ = 0.962
16660 measured reflections
7595 independent reflections
5635 reflections with *I* > 2σ(*I*)
*R*_int_ = 0.030

#### Refinement

*R*[*F* ^2^ > 2σ(*F* ^2^)] = 0.042
*wR*(*F* ^2^) = 0.109
*S* = 1.03
7595 reflections
367 parameters
1 restraint
H-atom parameters constrained
Δρ_max_ = 0.33 e Å^−3^
Δρ_min_ = −0.25 e Å^−3^
Absolute structure: Flack (1983 ▶), with 3584 Friedel pairs
Flack parameter: 0.07 (7)

### 

Data collection: *APEX2* (Bruker, 2004 ▶); cell refinement: *SAINT* (Bruker, 2004 ▶); data reduction: *SAINT*; program(s) used to solve structure: *SIR92* (Altomare *et al.*, 1993 ▶); program(s) used to refine structure: *SHELXL97* (Sheldrick, 2008 ▶); molecular graphics: *PLATON* (Spek, 2003 ▶); software used to prepare material for publication: *SHELXL97*.

## Supplementary Material

Crystal structure: contains datablocks I, global. DOI: 10.1107/S1600536808032108/bt2802sup1.cif

Structure factors: contains datablocks I. DOI: 10.1107/S1600536808032108/bt2802Isup2.hkl

Additional supplementary materials:  crystallographic information; 3D view; checkCIF report

## Figures and Tables

**Table 1:** Hydrogen-bond geometry (Å, °)

*D*—H⋯*A*	*D*—H	H⋯*A*	*D*⋯*A*	*D*—H⋯*A*
N3—H3*A*⋯O5^i^	0.86	1.95	2.801 (3)	171
N6—H6*A*⋯O2^ii^	0.86	1.96	2.799 (3)	164
C25—H25*C*⋯O2^ii^	0.96	2.37	3.238 (4)	150

Symmetry codes: (i) 
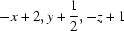
; (ii) 
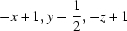
.

**Fig. 1.**
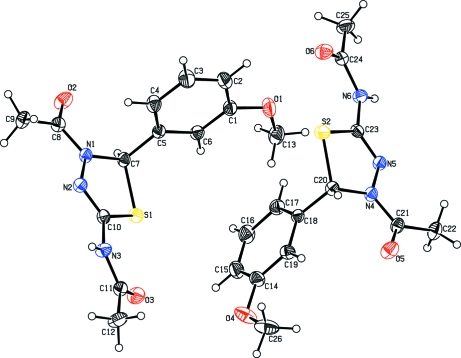
The molecular structure of the title compound, showing 30% probability displacement ellipsoids.

**Fig. 2.**
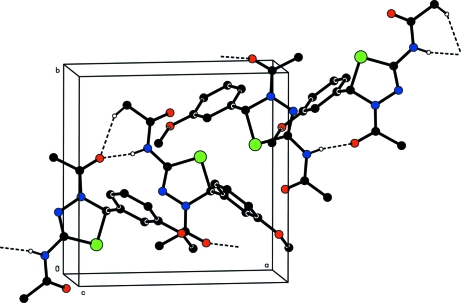
The molecular packing of the title compound. For clarity, hydrogen atoms which are not involved in hydrogen bonding were omitted.

## supplementary crystallographic information

### Comment

Nitrogen heterocycles are one of the most important classes of biologically active compounds. Suitably substituted 1,3,4-thiadiazoles have attracted great attention owing to their broad spectrum of biological activities in the areas of medicine which includes antimicrobial, antituberculosis, anesthetic, antithrombotic, anticonvulsant, antihypertensive, anti-inflammatory and antiulcer activities (Balasubramanian *et al.*, 2004; Li *et al.*, 2001; Radwan *et al.* 2007; Supuran *et al.*, 2001). Their action depends directly on the type and location of polar substituents on the heterocyclic ring. In general, pharmacological effect of potential drugs depends sensitively and solely on the stereochemistry and ring conformations. Thus, by keeping in view the promising biological potency of 1,3,4-thiadiazoles and variously substituted 1,3,4-thiadiazole frameworks, we have carried out the crystal structure determination of the title compound.

The title compound crystallizes with two molecules in the asymmetric unit. The sum of the angles at N1 (359.9 (6)°) and N4 (360.0 (6)°) are in accordance with *sp*^2^ hybridization. The torsion angles around C6—C1—O1—C13 [0.0 (4)°] and C19—C14—O4—C26 [-1.8 (4)°] indicates the coplanarity of the methoxy groups with the corresponding phenyl rings (C1–C6) and (C14–C19), respectively. The thiadiazole ring in both the molecules adopt envelope conformation with atoms C7 and C20 deviating by 0.395 (3) and 0.350 (3) Å, respectively, from the mean plane of the remaining atoms. The puckering parameters (Cremer & Pople, 1975) and the smallest displacement asymmetry parameters (Nardelli, 1983) for the thiadiazole rings C10—S2—C7—N1—N2 and C20—S2—C23—N5—N4 are q_2_ = 0.245 (2), 0.217 (2) Å, φ = 36.4 (6), 215.4 (6)° and Δ_s_(C_7_) = 6.9 (2), Δ_s_(C_20_) = 5.4 (2), respectively. N—H···O and C—H···O intermolecular interactions stabilize the crystal packing (Table 1).

### Experimental

The title compound was obtained by applying the method of Balasubramanian *et al.* (2004). 3-Methoxybenzadehyde thiosemicarbazone obtained by the reaction of 3-Methoxybenzadehyde and thiosemicarbazide was refluxed with excess of freshly distilled acetic anhydride on a water bath for about 7 h. After the completion of reaction, the excess of acetic anhydride was distilled off under reduced pressure and the obtained crude mass was purified by column chromatography (benzene–ethylacetate 5:1 *v*/*v*). Crystals were obtained from the solution of freshly distilled ethanol by slow evaporation at room temperature. ^1^H NMR (DMSO-*d*_6_, p.p.m.): 10.90 (s, 1H, amide NH); 7.36–6.76 (m, 5H, aromatic and ring methine protons); 3.78 (s, 3H, OCH_3_); 2.38 (s, 3H, –COCH_3_); 2.28 (s, 3H, –COCH_3_).

### Refinement

All H-atoms were refined using a riding model with d(C—H) = 0.93 Å, *U*_iso_ = 1.2*U*_eq_ (C) for aromatic, 0.98 Å, *U*_iso_ = 1.2*U*_eq_ (C) for CH, 0.96 Å, *U*_iso_ = 1.5*U*_eq_ (C) for CH_3_ and 0.86 Å, *U*_iso_ = 1.2*U*_eq_ (N) for NH atoms. The methyl groups were allowed to rotate but not to tip.

### Crystal data

**Table:**

C_13_H_15_N_3_O_3_S	*F*(000) = 616
*M**_r_* = 293.34	*D*_x_ = 1.422 Mg m^−^^3^
Monoclinic, *P*2_1_	Mo *K*α radiation, λ = 0.71073 Å
Hall symbol: P 2yb	Cell parameters from 5353 reflections
*a* = 11.3790 (4) Å	θ = 1.8–29.6°
*b* = 10.5993 (3) Å	µ = 0.25 mm^−^^1^
*c* = 11.9596 (2) Å	*T* = 293 K
β = 108.225 (2)°	Prism, colourless
*V* = 1370.08 (7) Å^3^	0.30 × 0.20 × 0.16 mm
*Z* = 4	

### Data collection

**Table:**

Bruker Kappa-APEXII CCD diffractometer	7595 independent reflections
Radiation source: fine-focus sealed tube	5635 reflections with *I* > 2σ(*I*)
graphite	*R*_int_ = 0.030
ω and φ scans	θ_max_ = 29.6°, θ_min_ = 1.8°
Absorption correction: multi-scan (SADABS; Bruker, 1999)	*h* = −15→15
*T*_min_ = 0.930, *T*_max_ = 0.962	*k* = −14→14
16660 measured reflections	*l* = −14→16

### Refinement

**Table:**

Refinement on *F*^2^	Secondary atom site location: difference Fourier map
Least-squares matrix: full	Hydrogen site location: inferred from neighbouring sites
*R*[*F*^2^ > 2σ(*F*^2^)] = 0.042	H-atom parameters constrained
*wR*(*F*^2^) = 0.109	*w* = 1/[σ^2^(*F*_o_^2^) + (0.0466*P*)^2^ + 0.2666*P*] where *P* = (*F*_o_^2^ + 2*F*_c_^2^)/3
*S* = 1.03	(Δ/σ)_max_ = 0.001
7595 reflections	Δρ_max_ = 0.33 e Å^−^^3^
367 parameters	Δρ_min_ = −0.25 e Å^−^^3^
1 restraint	Absolute structure: Flack (1983), with 3584 Friedel pairs
Primary atom site location: structure-invariant direct methods	Flack parameter: 0.07 (7)

### Special details

**Table:**

Geometry. All e.s.d.'s (except the e.s.d. in the dihedral angle between two l.s. planes) are estimated using the full covariance matrix. The cell e.s.d.'s are taken into account individually in the estimation of e.s.d.'s in distances, angles and torsion angles; correlations between e.s.d.'s in cell parameters are only used when they are defined by crystal symmetry. An approximate (isotropic) treatment of cell e.s.d.'s is used for estimating e.s.d.'s involving l.s. planes.
Refinement. Refinement of *F*^2^ against ALL reflections. The weighted *R*-factor *wR* and goodness of fit *S* are based on *F*^2^, conventional *R*-factors *R* are based on *F*, with *F* set to zero for negative *F*^2^. The threshold expression of *F*^2^ > σ(*F*^2^) is used only for calculating *R*-factors(gt) *etc*. and is not relevant to the choice of reflections for refinement. *R*-factors based on *F*^2^ are statistically about twice as large as those based on *F*, and *R*- factors based on ALL data will be even larger.

### Fractional atomic coordinates and isotropic or equivalent isotropic displacement parameters (Å^2^)

**Table:**

	*x*	*y*	*z*	*U*_iso_*/*U*_eq_	
C1	0.5869 (2)	0.7772 (2)	0.4618 (2)	0.0397 (5)	
C2	0.6405 (3)	0.8511 (3)	0.3955 (3)	0.0498 (8)	
H2	0.6027	0.8595	0.3148	0.060*	
C3	0.7499 (3)	0.9119 (3)	0.4499 (2)	0.0538 (7)	
H3	0.7862	0.9615	0.4055	0.065*	
C4	0.8069 (2)	0.9005 (3)	0.5697 (2)	0.0442 (6)	
H4	0.8808	0.9428	0.6055	0.053*	
C5	0.7547 (2)	0.8268 (2)	0.6360 (2)	0.0309 (5)	
C6	0.64253 (18)	0.7665 (2)	0.5812 (2)	0.0350 (5)	
H6	0.6051	0.7186	0.6258	0.042*	
C7	0.8123 (2)	0.8112 (3)	0.7677 (2)	0.0324 (6)	
H7	0.7546	0.8419	0.8076	0.039*	
C8	0.9343 (2)	1.0017 (3)	0.8382 (2)	0.0363 (6)	
C9	1.0571 (2)	1.0645 (3)	0.8708 (3)	0.0441 (7)	
H9A	1.0464	1.1541	0.8742	0.066*	
H9B	1.0969	1.0455	0.8129	0.066*	
H9C	1.1074	1.0346	0.9464	0.066*	
C10	1.0096 (2)	0.6892 (3)	0.8300 (2)	0.0315 (6)	
C11	1.0879 (3)	0.4753 (3)	0.8707 (2)	0.0390 (6)	
C12	1.1970 (3)	0.3942 (3)	0.8773 (3)	0.0569 (8)	
H12A	1.1920	0.3658	0.7997	0.085*	
H12B	1.1975	0.3226	0.9267	0.085*	
H12C	1.2716	0.4420	0.9098	0.085*	
C13	0.4198 (2)	0.6425 (3)	0.4616 (3)	0.0548 (7)	
H13A	0.3971	0.6925	0.5186	0.082*	
H13B	0.4748	0.5763	0.5011	0.082*	
H13C	0.3468	0.6062	0.4074	0.082*	
C14	0.8900 (2)	0.2827 (3)	0.5333 (2)	0.0431 (6)	
C15	0.8399 (3)	0.3437 (3)	0.6089 (3)	0.0499 (8)	
H15	0.8734	0.3319	0.6897	0.060*	
C16	0.7400 (3)	0.4223 (3)	0.5648 (2)	0.0524 (7)	
H16	0.7053	0.4623	0.6162	0.063*	
C17	0.6906 (2)	0.4425 (2)	0.4454 (2)	0.0420 (6)	
H17	0.6236	0.4967	0.4164	0.050*	
C18	0.7410 (2)	0.3820 (3)	0.3691 (2)	0.0313 (5)	
C19	0.84171 (19)	0.3018 (2)	0.4136 (2)	0.0365 (5)	
H19	0.8764	0.2611	0.3626	0.044*	
C20	0.6864 (2)	0.3966 (3)	0.2384 (2)	0.0316 (5)	
H20	0.7459	0.3662	0.2005	0.038*	
C21	0.5698 (2)	0.2018 (3)	0.1704 (2)	0.0352 (6)	
C22	0.4489 (2)	0.1337 (3)	0.1313 (3)	0.0457 (7)	
H22A	0.4280	0.1056	0.1990	0.069*	
H22B	0.4554	0.0621	0.0844	0.069*	
H22C	0.3857	0.1895	0.0855	0.069*	
C23	0.4851 (2)	0.5119 (3)	0.1630 (2)	0.0307 (5)	
C24	0.4060 (2)	0.7251 (3)	0.1174 (2)	0.0371 (6)	
C25	0.2952 (2)	0.8055 (3)	0.1067 (3)	0.0520 (8)	
H25A	0.2616	0.8347	0.0271	0.078*	
H25B	0.3190	0.8766	0.1587	0.078*	
H25C	0.2340	0.7569	0.1275	0.078*	
C26	1.0400 (3)	0.1360 (3)	0.5113 (3)	0.0692 (9)	
H26A	1.0753	0.1935	0.4686	0.104*	
H26B	0.9772	0.0863	0.4568	0.104*	
H26C	1.1035	0.0814	0.5586	0.104*	
N1	0.92957 (17)	0.8777 (2)	0.81336 (18)	0.0319 (5)	
N2	1.03611 (17)	0.8057 (2)	0.8278 (2)	0.0339 (5)	
N3	1.10017 (18)	0.5994 (2)	0.8432 (2)	0.0383 (5)	
H3A	1.1689	0.6224	0.8336	0.046*	
N4	0.57049 (17)	0.3249 (2)	0.1927 (2)	0.0332 (5)	
N5	0.46128 (17)	0.3956 (2)	0.16790 (19)	0.0330 (5)	
N6	0.39184 (18)	0.6002 (2)	0.1401 (2)	0.0357 (5)	
H6A	0.3199	0.5754	0.1399	0.043*	
O1	0.47967 (16)	0.7197 (2)	0.39944 (16)	0.0550 (5)	
O2	0.83930 (16)	1.0588 (2)	0.8297 (2)	0.0525 (5)	
O3	0.99404 (17)	0.4378 (2)	0.88764 (19)	0.0514 (5)	
O4	0.98715 (18)	0.2046 (2)	0.58456 (19)	0.0676 (6)	
O5	0.66898 (16)	0.1464 (2)	0.18601 (19)	0.0513 (5)	
O6	0.50089 (16)	0.7653 (2)	0.10697 (18)	0.0487 (5)	
S1	0.85560 (5)	0.64828 (6)	0.81229 (6)	0.03785 (17)	
S2	0.63984 (5)	0.55696 (6)	0.18841 (6)	0.03857 (17)	

### Atomic displacement parameters (Å^2^)

**Table:**

	*U*^11^	*U*^22^	*U*^33^	*U*^12^	*U*^13^	*U*^23^
C1	0.0346 (10)	0.0384 (13)	0.0415 (13)	0.0002 (9)	0.0053 (10)	−0.0048 (10)
C2	0.0547 (16)	0.054 (2)	0.0347 (15)	−0.0045 (14)	0.0053 (13)	0.0004 (13)
C3	0.0597 (15)	0.0617 (19)	0.0407 (15)	−0.0191 (14)	0.0166 (12)	0.0057 (13)
C4	0.0404 (12)	0.0500 (15)	0.0426 (14)	−0.0140 (11)	0.0136 (11)	−0.0016 (12)
C5	0.0285 (10)	0.0297 (14)	0.0349 (13)	0.0004 (9)	0.0102 (10)	−0.0034 (11)
C6	0.0299 (9)	0.0337 (12)	0.0405 (12)	−0.0024 (8)	0.0099 (9)	−0.0001 (10)
C7	0.0236 (10)	0.0341 (14)	0.0378 (14)	−0.0022 (10)	0.0071 (10)	−0.0053 (12)
C8	0.0347 (12)	0.0371 (16)	0.0382 (15)	0.0007 (11)	0.0129 (11)	−0.0028 (12)
C9	0.0377 (12)	0.0392 (16)	0.0552 (18)	−0.0079 (13)	0.0142 (12)	−0.0115 (15)
C10	0.0253 (10)	0.0370 (16)	0.0324 (13)	−0.0024 (10)	0.0093 (9)	−0.0015 (11)
C11	0.0412 (13)	0.0371 (16)	0.0370 (15)	−0.0039 (12)	0.0096 (11)	−0.0004 (12)
C12	0.0521 (16)	0.0326 (16)	0.086 (2)	0.0014 (14)	0.0209 (16)	0.0069 (17)
C13	0.0384 (12)	0.0568 (17)	0.0667 (18)	−0.0103 (12)	0.0129 (12)	−0.0108 (15)
C14	0.0331 (10)	0.0456 (15)	0.0455 (14)	−0.0001 (10)	0.0048 (10)	0.0045 (11)
C15	0.0554 (16)	0.055 (2)	0.0351 (16)	−0.0062 (14)	0.0080 (13)	−0.0022 (13)
C16	0.0600 (15)	0.0552 (18)	0.0441 (15)	0.0019 (13)	0.0192 (13)	−0.0117 (13)
C17	0.0406 (11)	0.0386 (13)	0.0458 (14)	0.0050 (10)	0.0119 (11)	−0.0053 (11)
C18	0.0251 (10)	0.0291 (14)	0.0374 (14)	−0.0036 (9)	0.0066 (10)	−0.0007 (11)
C19	0.0301 (9)	0.0380 (12)	0.0417 (13)	0.0036 (9)	0.0118 (9)	0.0018 (11)
C20	0.0237 (10)	0.0339 (14)	0.0383 (14)	−0.0015 (10)	0.0112 (10)	−0.0012 (12)
C21	0.0325 (12)	0.0351 (15)	0.0398 (15)	−0.0032 (11)	0.0141 (10)	−0.0071 (12)
C22	0.0401 (13)	0.0432 (18)	0.0551 (18)	−0.0099 (13)	0.0167 (12)	−0.0118 (15)
C23	0.0268 (10)	0.0343 (15)	0.0289 (13)	−0.0005 (10)	0.0056 (9)	0.0035 (10)
C24	0.0371 (13)	0.0330 (15)	0.0358 (14)	−0.0013 (11)	0.0036 (11)	0.0008 (12)
C25	0.0428 (14)	0.0347 (16)	0.074 (2)	0.0065 (12)	0.0111 (14)	0.0046 (16)
C26	0.0557 (16)	0.066 (2)	0.088 (2)	0.0258 (16)	0.0253 (16)	0.0328 (19)
N1	0.0219 (9)	0.0343 (13)	0.0381 (12)	−0.0010 (8)	0.0074 (8)	−0.0054 (10)
N2	0.0238 (9)	0.0348 (13)	0.0425 (13)	0.0014 (9)	0.0096 (9)	−0.0019 (10)
N3	0.0308 (10)	0.0327 (13)	0.0541 (14)	−0.0009 (8)	0.0171 (9)	0.0045 (10)
N4	0.0234 (9)	0.0334 (13)	0.0407 (13)	−0.0005 (9)	0.0069 (8)	−0.0048 (10)
N5	0.0257 (9)	0.0332 (13)	0.0388 (12)	0.0007 (9)	0.0083 (8)	−0.0038 (10)
N6	0.0272 (9)	0.0322 (13)	0.0461 (12)	−0.0002 (8)	0.0089 (8)	0.0034 (10)
O1	0.0433 (9)	0.0665 (13)	0.0459 (10)	−0.0145 (9)	0.0006 (8)	−0.0070 (10)
O2	0.0383 (9)	0.0388 (12)	0.0845 (15)	0.0039 (10)	0.0252 (10)	−0.0079 (12)
O3	0.0465 (11)	0.0472 (14)	0.0617 (13)	−0.0102 (10)	0.0187 (10)	0.0077 (11)
O4	0.0572 (11)	0.0840 (16)	0.0567 (12)	0.0303 (11)	0.0110 (10)	0.0231 (12)
O5	0.0363 (9)	0.0405 (12)	0.0795 (14)	0.0048 (10)	0.0216 (9)	−0.0079 (12)
O6	0.0431 (10)	0.0430 (13)	0.0617 (13)	−0.0049 (9)	0.0187 (9)	0.0097 (11)
S1	0.0281 (3)	0.0403 (4)	0.0434 (4)	−0.0051 (3)	0.0087 (2)	0.0057 (3)
S2	0.0264 (3)	0.0371 (4)	0.0505 (4)	−0.0023 (3)	0.0096 (3)	0.0090 (3)

### Geometric parameters (Å, °)

**Table:**

C1—O1	1.359 (3)	C14—C19	1.377 (3)
C1—C6	1.374 (3)	C15—C16	1.374 (4)
C1—C2	1.384 (4)	C15—H15	0.9300
C2—C3	1.372 (4)	C16—C17	1.378 (4)
C2—H2	0.9300	C16—H16	0.9300
C3—C4	1.380 (4)	C17—C18	1.378 (3)
C3—H3	0.9300	C17—H17	0.9300
C4—C5	1.374 (3)	C18—C19	1.392 (3)
C4—H4	0.9300	C18—C20	1.499 (3)
C5—C6	1.394 (3)	C19—H19	0.9300
C5—C7	1.514 (4)	C20—N4	1.471 (3)
C6—H6	0.9300	C20—S2	1.824 (3)
C7—N1	1.456 (3)	C20—H20	0.9800
C7—S1	1.829 (3)	C21—O5	1.234 (3)
C7—H7	0.9800	C21—N4	1.332 (4)
C8—O2	1.215 (3)	C21—C22	1.493 (3)
C8—N1	1.345 (4)	C22—H22A	0.9600
C8—C9	1.486 (4)	C22—H22B	0.9600
C9—H9A	0.9600	C22—H22C	0.9600
C9—H9B	0.9600	C23—N5	1.267 (4)
C9—H9C	0.9600	C23—N6	1.377 (3)
C10—N2	1.274 (3)	C23—S2	1.757 (2)
C10—N3	1.375 (3)	C24—N6	1.370 (4)
C10—S1	1.753 (2)	C24—C25	1.493 (4)
C11—O3	1.215 (3)	C25—H25A	0.9600
C11—N3	1.373 (4)	C25—H25B	0.9600
C11—C12	1.492 (4)	C25—H25C	0.9600
C12—H12A	0.9600	C26—O4	1.410 (4)
C12—H12B	0.9600	C26—H26A	0.9600
C12—H12C	0.9600	C26—H26B	0.9600
C13—O1	1.416 (3)	C26—H26C	0.9600
C13—H13A	0.9600	N1—N2	1.397 (3)
C13—H13B	0.9600	N3—H3A	0.8600
C13—H13C	0.9600	N4—N5	1.401 (3)
C14—O4	1.364 (3)	N6—H6A	0.8600
C14—C15	1.373 (4)		
			
O1—C1—C6	125.1 (2)	C16—C17—C18	119.6 (2)
O1—C1—C2	114.9 (2)	C16—C17—H17	120.2
C6—C1—C2	120.0 (2)	C18—C17—H17	120.2
C3—C2—C1	119.4 (3)	C17—C18—C19	119.6 (2)
C3—C2—H2	120.3	C17—C18—C20	121.3 (2)
C1—C2—H2	120.3	C19—C18—C20	119.0 (2)
C2—C3—C4	120.9 (3)	C14—C19—C18	120.0 (2)
C2—C3—H3	119.6	C14—C19—H19	120.0
C4—C3—H3	119.6	C18—C19—H19	120.0
C5—C4—C3	120.1 (2)	N4—C20—C18	111.4 (2)
C5—C4—H4	120.0	N4—C20—S2	103.01 (16)
C3—C4—H4	120.0	C18—C20—S2	114.9 (2)
C4—C5—C6	119.2 (2)	N4—C20—H20	109.1
C4—C5—C7	122.5 (2)	C18—C20—H20	109.1
C6—C5—C7	118.3 (2)	S2—C20—H20	109.1
C1—C6—C5	120.5 (2)	O5—C21—N4	119.2 (2)
C1—C6—H6	119.8	O5—C21—C22	121.8 (3)
C5—C6—H6	119.8	N4—C21—C22	118.9 (2)
N1—C7—C5	112.6 (2)	C21—C22—H22A	109.5
N1—C7—S1	102.46 (16)	C21—C22—H22B	109.5
C5—C7—S1	113.33 (19)	H22A—C22—H22B	109.5
N1—C7—H7	109.4	C21—C22—H22C	109.5
C5—C7—H7	109.4	H22A—C22—H22C	109.5
S1—C7—H7	109.4	H22B—C22—H22C	109.5
O2—C8—N1	119.7 (3)	N5—C23—N6	120.6 (2)
O2—C8—C9	122.5 (3)	N5—C23—S2	118.25 (19)
N1—C8—C9	117.7 (2)	N6—C23—S2	121.2 (2)
C8—C9—H9A	109.5	O6—C24—N6	121.8 (3)
C8—C9—H9B	109.5	O6—C24—C25	123.3 (3)
H9A—C9—H9B	109.5	N6—C24—C25	114.9 (2)
C8—C9—H9C	109.5	C24—C25—H25A	109.5
H9A—C9—H9C	109.5	C24—C25—H25B	109.5
H9B—C9—H9C	109.5	H25A—C25—H25B	109.5
N2—C10—N3	120.0 (2)	C24—C25—H25C	109.5
N2—C10—S1	118.12 (19)	H25A—C25—H25C	109.5
N3—C10—S1	121.8 (2)	H25B—C25—H25C	109.5
O3—C11—N3	120.9 (3)	O4—C26—H26A	109.5
O3—C11—C12	124.3 (3)	O4—C26—H26B	109.5
N3—C11—C12	114.8 (3)	H26A—C26—H26B	109.5
C11—C12—H12A	109.5	O4—C26—H26C	109.5
C11—C12—H12B	109.5	H26A—C26—H26C	109.5
H12A—C12—H12B	109.5	H26B—C26—H26C	109.5
C11—C12—H12C	109.5	C8—N1—N2	122.2 (2)
H12A—C12—H12C	109.5	C8—N1—C7	121.6 (2)
H12B—C12—H12C	109.5	N2—N1—C7	116.1 (2)
O1—C13—H13A	109.5	C10—N2—N1	109.3 (2)
O1—C13—H13B	109.5	C11—N3—C10	124.2 (2)
H13A—C13—H13B	109.5	C11—N3—H3A	117.9
O1—C13—H13C	109.5	C10—N3—H3A	117.9
H13A—C13—H13C	109.5	C21—N4—N5	122.2 (2)
H13B—C13—H13C	109.5	C21—N4—C20	122.0 (2)
O4—C14—C15	115.8 (2)	N5—N4—C20	115.8 (2)
O4—C14—C19	124.1 (2)	C23—N5—N4	110.0 (2)
C15—C14—C19	120.2 (2)	C24—N6—C23	124.4 (2)
C14—C15—C16	119.7 (3)	C24—N6—H6A	117.8
C14—C15—H15	120.1	C23—N6—H6A	117.8
C16—C15—H15	120.1	C1—O1—C13	117.9 (2)
C15—C16—C17	120.9 (3)	C14—O4—C26	118.5 (2)
C15—C16—H16	119.6	C10—S1—C7	88.3 (1)
C17—C16—H16	119.6	C23—S2—C20	88.6 (1)
			
O1—C1—C2—C3	−179.1 (3)	N3—C10—N2—N1	179.8 (2)
C6—C1—C2—C3	0.8 (4)	S1—C10—N2—N1	1.3 (3)
C1—C2—C3—C4	−0.1 (5)	C8—N1—N2—C10	162.3 (3)
C2—C3—C4—C5	0.5 (5)	C7—N1—N2—C10	−18.8 (3)
C3—C4—C5—C6	−1.4 (4)	O3—C11—N3—C10	−1.5 (4)
C3—C4—C5—C7	−179.6 (3)	C12—C11—N3—C10	178.4 (3)
O1—C1—C6—C5	178.2 (2)	N2—C10—N3—C11	166.0 (3)
C2—C1—C6—C5	−1.8 (4)	S1—C10—N3—C11	−15.5 (4)
C4—C5—C6—C1	2.1 (4)	O5—C21—N4—N5	174.7 (2)
C7—C5—C6—C1	−179.6 (2)	C22—C21—N4—N5	−7.5 (4)
C4—C5—C7—N1	−2.5 (4)	O5—C21—N4—C20	−1.6 (4)
C6—C5—C7—N1	179.3 (2)	C22—C21—N4—C20	176.2 (2)
C4—C5—C7—S1	−118.2 (2)	C18—C20—N4—C21	−81.9 (3)
C6—C5—C7—S1	63.5 (3)	S2—C20—N4—C21	154.4 (2)
O4—C14—C15—C16	−178.6 (3)	C18—C20—N4—N5	101.6 (3)
C19—C14—C15—C16	1.1 (4)	S2—C20—N4—N5	−22.2 (3)
C14—C15—C16—C17	−1.1 (5)	N6—C23—N5—N4	−179.8 (2)
C15—C16—C17—C18	0.8 (4)	S2—C23—N5—N4	−0.8 (3)
C16—C17—C18—C19	−0.4 (4)	C21—N4—N5—C23	−160.5 (3)
C16—C17—C18—C20	177.1 (3)	C20—N4—N5—C23	16.0 (3)
O4—C14—C19—C18	179.0 (2)	O6—C24—N6—C23	5.9 (4)
C15—C14—C19—C18	−0.7 (4)	C25—C24—N6—C23	−174.3 (2)
C17—C18—C19—C14	0.4 (4)	N5—C23—N6—C24	−170.8 (3)
C20—C18—C19—C14	−177.2 (2)	S2—C23—N6—C24	10.3 (4)
C17—C18—C20—N4	−74.4 (3)	C6—C1—O1—C13	0.0 (4)
C19—C18—C20—N4	103.1 (2)	C2—C1—O1—C13	180.0 (2)
C17—C18—C20—S2	42.2 (3)	C15—C14—O4—C26	177.9 (3)
C19—C18—C20—S2	−140.2 (2)	C19—C14—O4—C26	−1.8 (4)
O2—C8—N1—N2	−176.4 (2)	N2—C10—S1—C7	11.6 (2)
C9—C8—N1—N2	6.0 (4)	N3—C10—S1—C7	−166.9 (2)
O2—C8—N1—C7	4.7 (4)	N1—C7—S1—C10	−18.96 (18)
C9—C8—N1—C7	−172.9 (2)	C5—C7—S1—C10	102.70 (18)
C5—C7—N1—C8	82.3 (3)	N5—C23—S2—C20	−10.6 (2)
S1—C7—N1—C8	−155.5 (2)	N6—C23—S2—C20	168.4 (2)
C5—C7—N1—N2	−96.6 (3)	N4—C20—S2—C23	16.77 (18)
S1—C7—N1—N2	25.5 (3)	C18—C20—S2—C23	−104.62 (18)

### Hydrogen-bond geometry (Å, °)

**Table:**

*D*—H···*A*	*D*—H	H···*A*	*D*···*A*	*D*—H···*A*
N3—H3A···O5^i^	0.86	1.95	2.801 (3)	171
N6—H6A···O2^ii^	0.86	1.96	2.799 (3)	164
C25—H25C···O2^ii^	0.96	2.37	3.238 (4)	150

Symmetry codes: (i) −*x*+2, *y*+1/2, −*z*+1; (ii) −*x*+1, *y*−1/2, −*z*+1.
